# Serum zinc values, ankle brachial index and mortality in hemodialysis patients

**DOI:** 10.1186/s12882-022-02982-6

**Published:** 2022-11-04

**Authors:** Knehtl Maša, Piko Nejc, Ekart Robert, Hojs Radovan, Bevc Sebastjan

**Affiliations:** 1 Department of Nephrology, Clinic of Internal Medicine, University Medical Center Maribor, Maribor, Slovenia; 2 Faculty of Medicine, University of Maribor, Maribor, Slovenia; 3 Department of Dialysis, Clinic of Internal Medicine, University Medical Center Maribor, Maribor, Slovenia

**Keywords:** Zinc, Atherosclerosis, Oxidative stress, Chronic renal insufficiency, Haemodialysis

## Abstract

**Introduction:**

The atherosclerotic state of haemodialysis (HD) patients may be influenced by heavy metals. The purpose of our study was to assess the relationship between serum zinc (Zn) ankle brachial index (ABI) as a non-invasive diagnostic tool for atherosclerosis, and mortality in chronic haemodialysis (HD) patients.

**Methods:**

Sixty one HD patients were included (mean age 61.2 ± 13.8 years). The ABI was measured with an automated measuring device (ABPI MD, MESI®, Slovenia). Two groups of patients were formed based on the median value of Zn (14.1 mcmol/l). The average observation time was 2.8 years. Comorbidities (arterial hypertension (AH), diabetes mellitus (DM), dyslipidaemia), smoking and oral nutritional supplements (ONS) consumption were noted. Survival rates were analysed by Kaplan–Meier and Cox regression was used to determine the influence of Zn, ABI, AH, DM, dyslipidaemia, smoking and ONS.

**Results:**

Zn values were between 9.2 and 23.5 mcmol/l (14.4 ± 2.34), ABI values ranged from 0.8 to 1.4 (1.14 ± 0.12). Patients with lower Zn values had lower ABI (*p* = 0.036). Mean survival time of patients with higher Zn values was 985 days ± 277 days and with lower Zn values 1055 ± 143 days. Six (19.4%) patients with lower Zn and five (16.7%) patients with higher Zn died. We found statistically insignificant lower survival in patients with higher Zn. We failed to find any predictor of all-cause mortality, except for ONS consumption (95% CI 1.6–33.3; *p* = 0.012).

**Conclusions:**

Lower Zn is associated with lower ABI in HD patients, but we found no impact of Zn on patient survival.

## Introduction

End-stage renal disease (ESRD) is an important public health problem due to its high morbidity and mortality as well as social and financial consequences [1]. Many factors contribute to the morbidity and mortality risks of haemodialysis (HD) patients including protein-energy wasting, chronic inflammation, oxidative stress, and impaired immune system [2]. The most common cause of death in patients with ESRD include advanced atherosclerosis and cardiovascular events, which can be attributed to the culmination of traditional and non-traditional chronic kidney disease (CKD)-specific risk factors, such as chronic inflammation and oxidative stress, which are especially frequent in HD patients [1]. Long-term HD has significantly lowered plasma or serum zinc (Zn) concentrations [2], predisposing them to such complications. Zn is an essential trace element, which plays important roles in gene expression, protein synthesis and immune function [3]. Zn deficiency in HD patients may lead to elevated oxidative stress [3]. Increased oxidative stress leads to atherosclerosis, resulting in endothelial dysfunction, disturbed nitric oxide (NO) and nuclear factor (NF)-kappa B-related signalling, and to oxidative changes of low-density lipoprotein (LDL). Zn is enrolled in all these aspects mainly through its antioxidant and anti-inflammatory effects [4]. Consequently, Zn is involved in atherosclerotic process and its lack may be a strong risk factor for advanced atherosclerosis [4].

In the retrospective study of Koyama A et al. Zn deficiency in patients with critical limb ischemia was associated with lower rates of limb salvage, amputation free survival, and wound healing [5].

In the study of Tsuruoka T et al. they found that Zn deficiency impaired the rate of ischemia-induced revascularization through enhanced oxidative stress rates in animal model. What is more, the skin perfusion pressures were positively associated with the serum zinc levels in patients with chronic limb-threatening ischemia. The authors concluded that circulating Zn levels could be a useful marker for the assessment of atherosclerosis-based vascular disease such as limb ischemia [6].

Peripheral arterial disease (PAD) can be an example of atherosclerotic process that couples structural and functional changes of the vessel wall [7]. A reduction in diameter of the arterial lumen is a major structural change and can be determined by using the ankle-brachial index (ABI). ABI has several important prognostic implications as it can be a sign of atherosclerosis in other vascular territories, especially coronary arteries [8, 9].

The goal of our study was to assess the relationship between serum Zn values, ABI as a non-invasive diagnostic tool for atherosclerosis, and mortality in chronic HD patients. To our best knowledge such a connection has not been studied before.

## Materials and methods

### General design of the study

This cohort study was held at the Department of Dialysis of the Maribor University Medical Centre, Slovenia. Sixty-one chronic HD patients were included in our study (mean age 61.2 ± 13.8 years, range 30 to 85 years), who received regular dialysis for at least 6 months. Patients after lower limb amputation were not included. With all patients, four-hour post-dilution hemodiafiltration (HDF) procedure three times weekly with a high-flux dialyzer was performed. Comorbidities (arterial hypertension (AH), diabetes mellitus (DM), dyslipidaemia), smoking and the use of oral nutritional supplements (ONS) were recorded. Patients were receiving two different ONS, which had been prescribed according to serum albumin level below 40 g/l: Fresubin**,** which contains 1.5 mg/100 ml of Zn sulphate and Abbott Nepro HP, which contains 1.9 mg/100 ml of Zn sulphate. ONS were prescribed as one dosage daily.

The observation time was from 5 September 2017 until death or 5 September 2020. The average observation time was 2.8 years.

Research was performed in accordance with the guidelines of the Declaration of Helsinki and informed consent was obtained from all participants. Research was performed according to Good Clinical Practice standards and was approved by the National Ethics Committee (N°0120–32/2017/4).

### Ankle-brachial index measurements

ABI was measured by a non-invasive, previously validated [10] automated measuring device (ABPI MD, MESI®, Slovenia), based on oscillometry. Measurements were performed after the participant had been lying still for five minutes. Blood pressure was measured in the supine position at the same time on the brachial part of the arm without arterio-venous fistula and calves of both legs. All BI measurements were done by the same person in all the patients [10]. ABI values < 0.90 were considered to be significant for peripheral artery disease (PAD), values > 1.40 referred to incompressible ankle arteries and values in between were considered as normal [10].

### Blood samples

All blood samples were obtained prior to HDF sessions and were taken from arterio-venous fistulas. Plasma concentrations of serum C-reactive protein (CRP), serum albumin, serum phosphorus, total cholesterol, as well as triglycerides in predialysis blood were recorded. Serum Zn values were measured by standard laboratory methods, the normal value of Zn in our laboratory is 10.0–19.7 mcmol/l.

### Statistical analysis

The statistical analysis was implemented with the Statistical Package for Social Sciences, version 22.0 (SPSS Inc, Chicago, IL, USA). Basic descriptive statistics were used for continuous variables (mean ± standard deviation (SD)). For categorical variables, frequencies and percentages were used. Patients were divided in two groups based on the median Zn value, unpaired t-test and Chi-squared test for categorical variables were used for between group comparisons. Survival rates were analysed using Kaplan–Meier survival curves. The Cox regression was used to assess the influence of Zn, ABI, AH, DM, dyslipidaemia, smoking and ONS consumption on all-cause mortality.

## Results

**Fig. 1:**
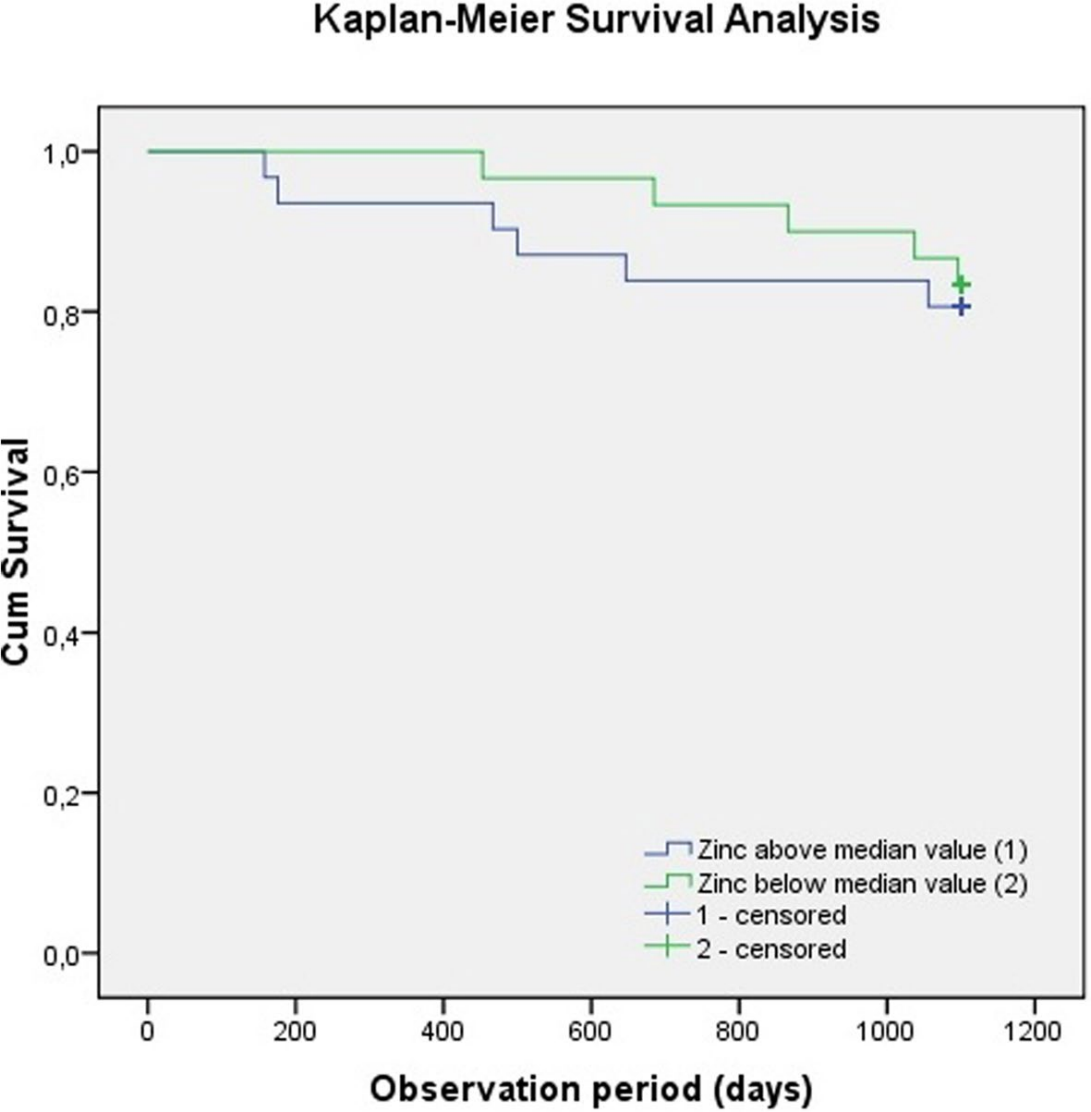
Kaplan–Meier Survival Analysis for different Zinc values

Serum Zn values were between 9.2 and 23.5 mcmol/l. The mean value of Zn was 14.4 ± 2.34 mcmol/L. Only one patient had serum Zn level below normal and only one patient had serum Zn level above normal. Two groups of patients were formed based on median value of Zn (14.1 mcmol/l) as a cut-off. Table 1 shows the baseline characteristics of patients – the group of patients with higher levels of serum Zn (≥ 14.1 mcmol/l) and the group of patients with lower levels of serum Zn (< 14.1 mcmol/l), their comorbidities and baseline laboratory results. The value of serum albumin was statistically significantly higher in the group of patients with a higher serum Zn level. We found that more than 40% of our patients were using ONS continuously and independently from Zn values. We found no statistically significant difference between the two groups in ONS consumption, although in the group of patients with serum Zn level above the median value, the percentage of patients taking ONS was higher, which was not statistically significant. ABI values ranged from 0.8 to 1.4, the mean value of ABI was 1.14 ± 0.12. A statistically significant difference in ABI values between the two groups was found (*p* = 0.036). Patients with lower Zn values had lower ABI. The mean survival time of patients with higher Zn values was 985 ± 277 days and with lower Zn values was 1055 ± 143 days. Six (19.4%) patients with lower Zn values and five (16.7%) patients with higher Zn values died. Lower survival for patients with higher Zn values in the observed period was shown by the Kaplan–Meier survival analysis, but the finding was not statistically significant (Fig. 1). Bivariate analysis showed statistically significant correlation to mortality for ONS consumption only (*p* = 0.0032). Moreover, following bivariate analysis in the Cox univariable and multivariable regression model we failed to find any predictors of all-cause mortality in our patients, except for ONS consumption (Hazard ratio (HR) 7.2; 95% confidence interval (CI) (1.6–33.3); *p* = 0.012) (Table 2).

**Table 1:** Baseline characteristics of HD patients with serum Zn below and above median value

Parameter	Serum zinc below median value (*n* = 30)*	**Serum zinc above median value (*n* = 31)**^**#**^	***p* value**
Age (years)	63.3 ± 15.8	59.7 ± 11.0	0.311
Male gender (n)	53.3% (16)	54.8% (17)	0.695
Body Mass Index (BMI; kg/m^2^)	26.8 ± 5.2	25.0 ± 5.2	0.183
Ankle-brachial index (ABI)	1.2 ± 1.4	1.1 ± 1.0	0.036
Arterial hypertension (n)	90% (27)	74.3% (23)	0.111
Diabetes mellitus (n)	30% (9)	19.4% (6)	0.338
Smoking (n)	30% (9)	41.9% (13)	0.336
C-reactive protein – CRP (mg/L)	11.4 ± 19.7	5.7 ± 6.0	0.130
Serum albumin (g/L)	37.3 ± 5.2	40.5 ± 3.2	0.005
Serum phosphate (mmol/L)	1.4 ± 0.5	1.6 ± 0.5	0.097
Total cholesterol (mmol/L)	3.8 ± 0.8	4.3 ± 1.1	0.055
Triglycerides (mmol/L)	1.7 ± 0.7	1.7 ± 1.4	0.768
Oral nutritional supplement (n/%)	12/40	14/45.2	0.686

^*^^#^All patients enrolled in the study had four-hour post-dilution hemodiafiltration procedure three times weekly with equal high-flux dialyzers

**Table 2:** Cox univariable and multivariable regression model analysis

	Univariable model			Multivariable model		
**Variable**	**HR**	**95% CI of HR**	***p* value**	**HR**	**95% CI of HR**	***p* value**
Zinc	1.226	0.950 – 1.582	0.118	0.989	0.715 – 1.366	0.945
Ankle-brachial index	0.201	0.002 – 22.472	0.505	0.282	0.001 – 58.815	0.643
Arterial hypertension	0.979	0.212 – 4.534	0.979	0.589	0.111 – 3.128	0.535
Diabetes mellitus	3.547	0.454 – 27.717	0.227	5–474	0.544 – 55.050	0.149
Smoking	0.588	0.179 – 1.928	0.381	0.585	0.160 – 2.141	0.418
Total cholesterol	1.023	0.564 – 1.855	0.940	0.908	0.382 – 2.155	0.826
Triglycerides	1.364	0.865 – 2.153	0.182	1.630	0.787 – 3.378	0.189
Oral nutritional supplement	7.177	1.549 – 33.260	**0.012**	8.094	1.473–44.467	**0.016**

*HR* Hazard ratio, *Cl* Confidence interval; *p* value < 0.05 was considered statistically significant in all analysis

**Fig. 2:**
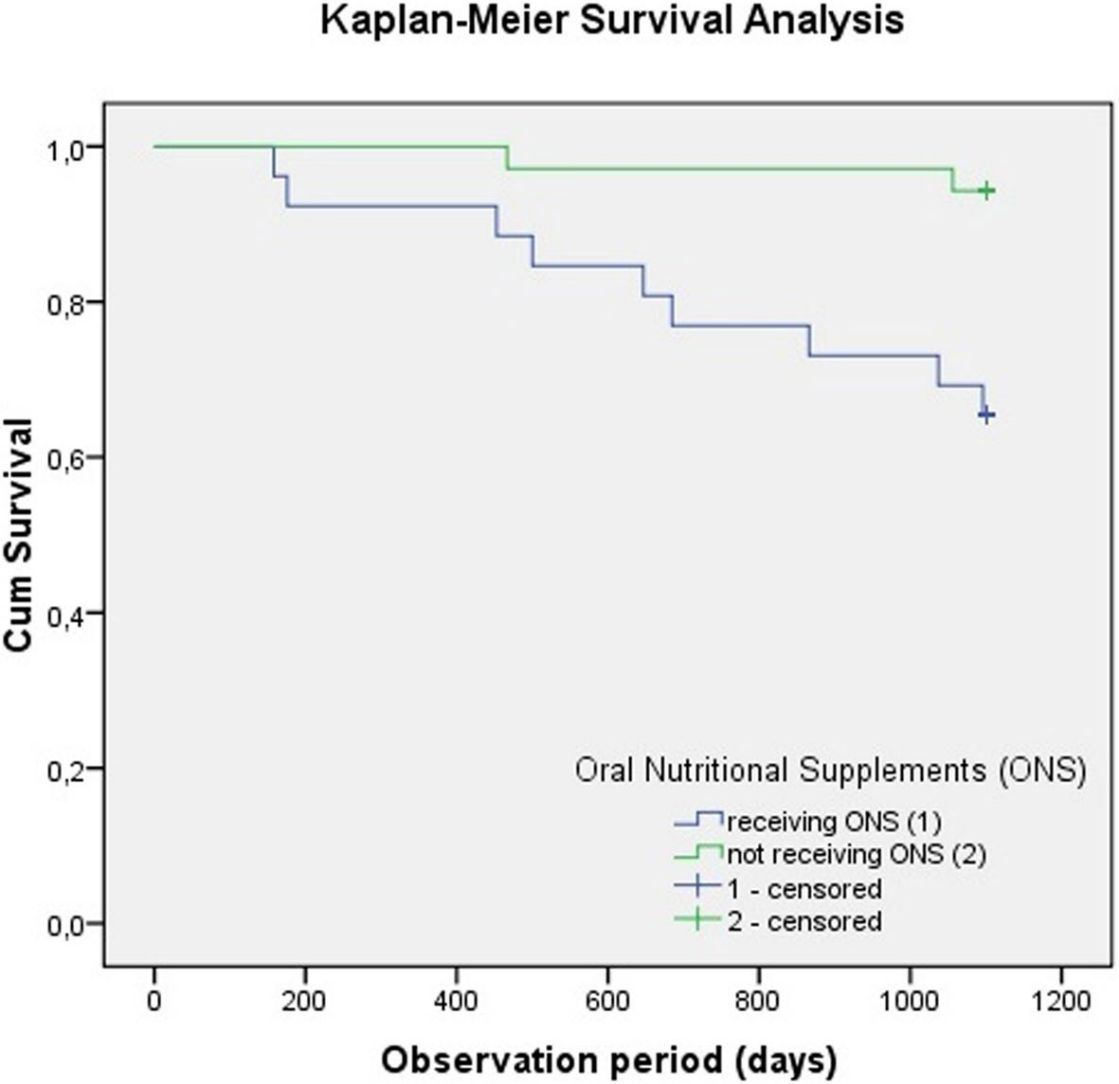
Kaplan–Meier Survival Analysis for patients with or without oral nutritional supplements

Additionally, a statistically significant higher risk of death (*p* = 0.003) was shown by the Kaplan–Meier survival analysis in patients who were receiving ONS (Fig. 2).

## Discussion

### Zinc and haemodialysis

Zn concentration has been accepted as a valid indicator of whole-body Zn in the absence of influencing factors such as infection or stress [11]. Zn deficiency in HD patients has a prevalence of 40–78% [12]. Only 1 out of 61 (1.6%) included dialysis patients had zinc deficiency in our study. An association between low serum Zn level and multiple protein energy malnutrition (PEM) parameters was proposed, which can predict poor clinical outcomes in chronic HD patients [12]. Low serum Zn concentrations could be a consequence of Zn removal by dialysis, low levels of albumin, and reduced absorption of Zn by gastrointestinal tract [2]. What is more, inflammation processes can reduce the plasma Zn levels [2]. Older HD patients had lower serum Zn levels than younger, however, a direct correlation between age and Zn level was not found [13]. In patients on peritoneal dialysis, Zn had a positive correlation with serum prealbumin level [13]. The same was shown in HD patients [12].

However, Zn deficiency was not present in our group of HD patients that can be explained by the fact that more than 40% of our patients used ONS continuously and independently from Zn values.

### Zinc and inflammation

Fifteen randomized controlled trials meta-analysis showed that supplementing Zn had anti-inflammatory and antioxidant effects in chronic HD patients [3]. The antioxidant activity of Zn involves two mechanisms connected with sulfhydryl and metallothionein [3]. Zn could also decrease CRP and other inflammatory cytokines by increased antioxidant activity, the major target is most likely the NF-kappa B-related signalling pathway [3]. It was found that Zn supplementation decreased the level of CRP in dialysis patients [14].

In our study no statistically significant difference in CRP values between the patients that had serum Zn level above or below the median value was found.

### Zinc and atherosclerosis

The progression of atherosclerosis is influenced by nutritional parameters including Zn. Zn deficiency could contribute to atherosclerotic process and there was an inverse relationship between atherosclerosis and serum Zn levels [4]. In the study conducted by *Yang *et al. the subclinical atherosclerosis was determined by using carotid intima-media thickness (cIMT) and analysed its relationship with the dietary Zn intake. In the middle-aged and elderly, lower Zn intake was associated with a higher cIMT than the higher Zn intake [15]. *Ari *et al. proposed that the copper/Zn ratio is an independent determinant of cIMT in chronic HD patients without known atherosclerosis. A negative correlation between cIMT and serum levels of Zn was found [16].

Despite the fact that ABI is known as a marker of atherosclerosis in haemodialysis patients [17], there is no study relating Zn and ABI in haemodialysis patients to our knowledge.

In our study, lower ABI was associated with lower Zn levels. Since lower ABI is a marker of atherosclerosis, it appears that in our patients lower Zn values relate to a higher risk of atherosclerosis.

### Zinc and lipids

Although studies suggested that Zn supplementation improves blood lipid metabolism, the effects of Zn supplementation are conflicting with several possible reasons mentioned [3, 12]. Zn supplementation could increase blood lipids in patients with anorexia and have an opposite effect in patients with hyperlipidaemia or insulin resistance [3]. Hypercholesterolemia and hypertriglyceridemia have been observed in patients with Zn-deficient diets, which could lead to cardiovascular complications and insulin resistance in CKD patients [3]. A positive correlation between Zn and serum total cholesterol concentrations in the malnourished HD patients has been shown [10]. A higher mortality of HD patients was found with predialysis cholesterol levels below 3.88 mmol/l [12]. Since HD patients are often Zn-deficient, Zn supplementation may also increase abnormally low serum lipid concentrations, connected with malnutrition and morbidity in the HD patients [12].

However, in our study the mortality was higher in the group of patients with serum Zn above the median value, and this group also had a higher total cholesterol level (although not statistically significant). The group of patients with lower Zn level had serum total cholesterol slightly above the minimum value for the above-mentioned increased mortality and the group of the patients with higher Zn level had normal total cholesterol.

### Zinc and mortality

The study of Yang et al. included 111 patients on maintenance HD, which were followed for 2 years or until their death. The authors concluded that in chronic dialysis patients, the serum level of Zn was an independent predictor of hospitalisations caused by infectious diseases and of all-cause mortality. They found that lower serum level of Zn independently predicts all-cause mortality, the mean serum value of Zn in the study was in the range of low normal [18]. In the study of Lobo et al., HD patients had low serum Zn levels, and high LDL and TNF-alpha levels compared to healthy subjects. Serum Zn levels correlated negatively with TNF-alpha and LDL, low Zn levels were associated with lipid peroxidation and inflammation and the inflammation markers were significant predictors of cardiovascular mortality [19]. Most above-mentioned studies are dealing with malnutrition, the lack of Zn and consequently higher mortality.

In our study, patients were already taking ONS that included Zn. Patients with higher levels of serum Zn had a higher risk of death, which was not statistically significant, however, our mean value of Zn was in normal range. Patients with higher Zn level had statistically significantly higher level of serum albumin, the percentage of patients taking ONS in this group was higher, however, the difference was not statistically significant. The difference in BMI between the two groups was not statistically significant. We propose that the decreased survival of patients with higher Zn values and ONS supplementation was not directly affected by these two parameters but was only a marker of previous malnourishment and generally worse condition of these patients (since the criteria for prescription was serum albumin level below 40 g/l). However, due to the retrospective design of the study, this is only a possible explanation and therefore further prospective research should be conducted to draw definitive conclusions.

### Study limitations

A small number of patients is a limitation to our study. Only Caucasian patients were included.

Substitution volume differences during HDF between the patients which could have had an effect on serum Zn level were not considered. We also did not factor in different high-flux membranes between patients and their individual properties, especially regarding micro-trace elements removal. The history of ONS consumption should have also been considered.

## Conclusions

In contrast to the results that we can find in literature, we have not confirmed Zn deficiency in our HD patients. Lower serum Zn was associated with lower ABI in HD patients, but no statistically significant impact of Zn values on patient survival was found. A possible explanation would be that decreased survival of the patients with higher Zn values and ONS supplementation was not directly affected by these two parameters but was only a marker of previous malnourishment and generally worse condition of these patients. Further prospective research is needed to define the role of zinc, atherosclerosis and mortality in ESRD.

## Data Availability

The datasets generated and/or analysed during the current study are not publicly available due to the policy of the institution but are available from the corresponding author on reasonable request.
