# Correction: Serum zinc values, ankle brachial index and mortality in hemodialysis patients

**DOI:** 10.1186/s12882-022-03032-x

**Published:** 2022-12-09

**Authors:** Maša Knehtl, Nejc Piko, Robert Ekart, Radovan Hojs, Sebastjan Bevc

**Affiliations:** 1 Department of Nephrology, Clinic of Internal Medicine, University Medical Center Maribor, Maribor, Slovenia; 2 Faculty of Medicine, University of Maribor, Maribor, Slovenia; 3 Department of Dialysis, Clinic of Internal Medicine, University Medical Center Maribor, Maribor, Slovenia

**Correction: BMC Nephrology (2022) 23:355**

**https://doi.org/10.1186/s12882-022-02982-6**

Following publication of the original article [1], we have been informed that first name and last name have been swapped for all the authors.

The author group has been updated above and the original article [1] has been corrected.
